# Disentangling controls on animal abundance: Prey availability, thermal habitat, and microhabitat structure

**DOI:** 10.1002/ece3.7930

**Published:** 2021-07-24

**Authors:** Emma A. Higgins, Doreen S. Boyd, Tom W. Brown, Sarah C. Owen, Adam C. Algar

**Affiliations:** ^1^ School of Geography University of Nottingham Nottingham UK; ^2^ Kanahau Utila Research and Conservation Facility Isla de Utila, Islas de Bahia Honduras; ^3^ Department of Biology Lakehead University Thunder Bay ON Canada

**Keywords:** abundance, *Anolis*, habitat structure, microhabitat, population size, prey availability, thermal ecology, tropical forests

## Abstract

The question of what controls animal abundance has always been fundamental to ecology, but given rapid environmental change, understanding the drivers and mechanisms governing abundance is more important than ever. Here, we determine how multidimensional environments and niches interact to determine population abundance along a tropical habitat gradient. Focusing on the endemic lizard *Anolis bicaorum* on the island of Utila (Honduras), we evaluate direct and indirect effects of three interacting niche axes on abundance: thermal habitat quality, structural habitat quality, and prey availability. We measured *A. bicaorum* abundance across a series of thirteen plots and used N‐mixture models and path analysis to disentangle direct and indirect effects of these factors. Results showed that thermal habitat quality and prey biomass both had positive direct effects on anole abundance. However, thermal habitat quality also influenced prey biomass, leading to a strong indirect effect on abundance. Thermal habitat quality was primarily a function of canopy density, measured as leaf area index (LAI). Despite having little direct effect on abundance, LAI had a strong overall effect mediated by thermal quality and prey biomass. Our results demonstrate the role of multidimensional environments and niche interactions in determining animal abundance and highlight the need to consider interactions between thermal niches and trophic interactions to understand variation in abundance, rather than focusing solely on changes in the physical environment.

## Graphical abstract

The question of what controls animal abundance has always been fundamental to ecology, but given rapid environmental change, understanding the drivers and mechanisms governing abundance is more important than ever. Here, we determine how multidimensional environments and niches interact to determine population abundance of an endemic lizard (*Anolis bicaorum*) along a habitat gradient in tropical forest by evaluating direct and indirect effects of three interacting niche axes on abundance: thermal habitat quality, structural habitat quality, and prey availability. Our findings show complex direct and indirect effects of these niche axes and highlight the importance of multidimensional environments and niche interactions in determining animal abundance.

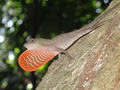

## 1 INTRODUCTION

The question of what determines population size is fundamental to ecology, biogeography, and conservation biology (Andrewartha & Birch, 1986; Brown, 1995; Lack, 1954). Complex intrinsic and extrinsic factors regulate population abundance (Pringle et al., 2019; Stapley et al., 2015), and classic and modern niche theory states that organisms are affected by multiple abiotic and biotic factors along multiple niche axes. These limit their abundance and distribution either by limiting resource availability directly or limiting species' ability to capture the resources that are available (Chase & Leibold, 2003). Identifying the factors responsible for population change along habitat gradients will improve our understanding of how multidimensional environments and niches interact to determine population abundance. Furthermore, conservation efforts and risk modeling can greatly benefit from isolating such mechanisms (Frishkoff et al., 2015).

While ecological niche theory is well developed, empirical evidence for which factors are most important, and how they interact, is still rare for many taxa. For example, *Anolis* lizards (anoles), our focus here, are a classic model system for evolutionary ecology and their behavior, morphology, physiology, microhabitat use, and evolutionary history have been extensively studied (reviewed in Losos, 2009). However, the question of what controls anole population size remains unanswered (Losos, 2009). Research by Buckley and Roughgarden (2005) and more recently by Frishkoff et al. (2019) have begun to address this gap, focusing on anole abundance and community structure along elevational gradients. Their work has indicated a role for canopy loss, thermal environment, changes in food resources, and competitive interactions in influencing animal abundance. However, the relative importance of these factors, and how they interact to influence abundance, remains unknown.

Niche theory tells us that abundance can be limited by abiotic and biotic factors, acting either from the bottom up or from the top down (Elton, 1927; Leroux & Loreau, 2015). Potential limiting factors include microclimate, structural microhabitat, food resource (prey) availability, competitors, mutualists, predators, parasites, and disease. For ectotherms, microclimate is expected to be especially important. Ectotherm body temperature (*T*_b_), which affects metabolic and ecological function and evolutionary fitness, is determined by the interaction between behavior, biophysics, and microclimate (Campbell & Norman, 1998; Gates, 1980; Huey & Slatkin, 1976). Unfavorable microclimatic conditions, that is, low thermal habitat quality, are predicted to restrict activity times, which in turn limits foraging, territory defense, and reproduction, leading to population declines (Sinervo et al., 2010). However, recent work has also suggested that anoles are often active in thermally sub optimal conditions, raising the possibility that thermal habitat quality may not exert as rigid controls on animal ecology, and thus population size, as traditionally thought (Gunderson & Leal, 2016; Méndez‐Galeano et al., 2020).

Changes in the suitability, extent, and complexity of structural microhabitat can potentially influence abundance. This may be especially true for semi‐arboreal and arboreal species, including most anoles, which have specific adaptations to increase performance in particular arboreal microhabitats (reviewed in Losos, 2009). For example, longer legs confer an advantage for increased running speed on broad substrates, whereas shorter limbs provide greater maneuverability on narrow surfaces (Kolbe & Losos, 2005). Given these well‐established microhabitat–ecology associations, perch availability is often used as an indicator of suitable habitat for anoles (e.g., Johnson et al., 2006). Changes in structural microhabitat, for example, perch structure and availability, can alter anole abundance in species‐specific ways (Frishkoff et al., 2019) and can select for phenotypic changes in urban anoles (Winchell et al., 2016). However, losses of suitable structural habitat do not occur in isolation and may be accompanied by altered prey communities and thermal conditions (Frishkoff et al., 2019), which in turn is mediated by changes in canopy cover (Algar et al., 2018).

Generally, predator biomass scales with prey biomass (Hatton et al., 2015). Loss of food resources, for example, climate‐induced declines in arthropod diversity and biomass, has been proposed to negatively affect the abundance of predators, including anoles (Lister & Garcia, 2018, but see Willig et al., 2019, Lister & Garcia, 2019). As with climate, changes in prey abundance may mediate impacts of other factors on abundance. For example, habitat alteration, such as urbanization, can have a negative effect on terrestrial arthropod diversity and abundance (Fenoglio et al., 2020) and therefore has the potential to negatively impact insectivore populations. Similarly, competition for prey may reduce the amount of resources captured by a species, an effect that could be exacerbated by introduced or invasive species that can reach high abundances, especially in modified habitats. For example, *Anolis sagrei*, a successful invader of urban and human‐modified environments (Kolbe et al., 2016), competes with native species, altering behavior and microhabitat use, and inducing evolutionary change (Kamath et al., 2013; Stroud et al., 2017; Stuart et al., 2014).

**FIGURE 1:**
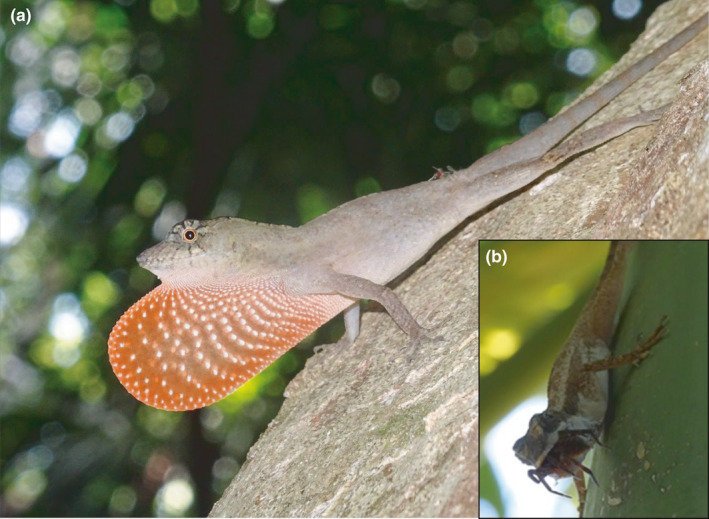
(a) Male *Anolis bicaorum* displaying dewlap, (b) *A. bicaorum* predating on an unidentified spider (*Araneae*)

Here, we ask what factors influence the abundance of the endemic lizard, *Anolis bicaorum* (Figure 1)*,* by considering multiple niche axes across gradients within tropical forest, on the island of Utila, Honduras. We focus on three niche axes potentially important for lizards: thermal habitat quality (Logan et al., 2013; Sears et al., 2016), structural habitat quality (Johnson et al., 2006), and prey availability (Battles et al., 2013), and use structural equation modeling to disentangle direct and indirect effects of these factors on *A. bicaorum* abundance.

## 2 MATERIALS AND METHODS

### 2.1 Study species and study sites

*Anolis bicaorum* is a small, predominantly arboreal lizard first described by Köhler (1996) endemic to Utila, Honduras. Males have an average snout–vent length (SVL) of approximately 64 mm SVL (McCranie & Köhler, 2015) and a bright orange‐red dewlap. Despite initial reports (McCranie & Köhler, 2015), females are smaller than males (average SVL = 62 mm) with a smaller dewlap that varies from cream/gray to red (White et al., 2019). *Anolis bicaorum* is thought to be a predominantly sit‐and‐wait predator that feeds primarily on arthropods (Brown, Maryon & Lonsdale, 2017) and descends to the ground at times in pursuit of prey (personal observation). It is found predominantly in forests (Brown, Maryon & Lonsdale, 2017), and its thermal ecology reflects these relatively cool, thermally homogeneous environments (Logan et al., 2013). *A. bicaorum* is one of two anoles endemic to Utila. The other, *Anolis utilensis*, is the only potential congeneric competitor of *A. bicaorum* in forests. However, despite being found in similar habitats, *A. utilensis* is found at much lower abundances and perches substantially higher in the canopy than *A. bicaorum* (Brown, Maryon, Van den Berg et al., 2017). Other anoles on the island include native *A*. *unilobatus*, which is found in open, grassy areas (McCranie & Orellana, 2014), the invasive species *A. sagrei,* which is presently restricted to Utila Town (Brown & Diotallevi, 2019), and records of *A. allisoni,* also from Utila Town, which likely reflect human introduction (Brown & Diotallevi, 2019).

Utila (16.0950°N, 86.9274°W) is one of the Bay Islands of Honduras, which host a number of marine and terrestrial protected areas under SINAPH (Honduras National System of Protected Areas) that are of local and international significance. It features a mosaic of habitats, including mangrove, tropical dry forest, Neotropical savanna, and volcanic rock exposures (Fawcett et al., 2016; Schulte & Köhler, 2010). The island is small, with a total area of 41 km^2^, with a single elevation gradient located toward the northeast Pumpkin Hill, with a maximum elevation of 74 m. The majority of the island varies from sea level to 8 m in elevation. We surveyed thirteen 20 × 20 m plots located toward the eastern portion of the island, where the majority of the forest is located. Plots varied in their level of human disturbance (personal observation), including relatively intact forest, to heavily disturbed, sparsely treed areas, in Utila Town. For a map of plot locations, please see Appendices S1 and S2.

### 2.2 Lizard surveys

We carried out abundance surveys for *A*. *bicaorum* using standard mark–recapture methods, based on Heckel and Roughgarden (1979). In each plot, the same observers actively searched for anoles for 60 min on four occasions (09:00, 13:00, 17:00, and 09:00), over a twenty‐five hour period. Each anole was marked with a visit‐specific paint mark using an Indico Duz‐all spray paint gun and nontoxic water‐based paint following Frishkoff et al. (2019), and Heckel and Roughgarden (1979). Plots were surveyed over a period of 10 weeks. We avoided days with rain or high winds, which may influence detectability and recorded air temperature at 1.5 m height using a shaded DS1921G‐F5 iButton at each plot.

### 2.3 Thermal environment

#### 2.3.1 Operative temperature

We measured operative temperature (*T*_e_) of lizards within different microhabitats in each plot. Twenty morphologically accurate 3D printed *Anolis* models, calibrated against a live lizard's body temperature (*T*_b_, see Appendices S1 and S2) and fitted with DS1921G‐F5 iButtons, were set up in each plot for three days. iButtons were programmed to record temperatures at 1‐hr intervals between the hours of 06:00 and 18:00, when anoles were active, giving a total sample period of 36 hr per plot. Model position, substrate (trunk vs. ground), height (0–250 cm in 15‐cm increments), and compass orientation (0–360° in 45° increments) were randomly chosen using a random number generator. Models were based on a 3D scan of a museum specimen and painted to match the coloration of *A*. *sagrei* as resources to base models on *A. bicaorum* were unavailable. We account for this in subsequent analyses by calibrating models against a live *A. bicaorum* individual. We carry out our analysis for the uncalibrated *T*_e_ data and two alternative calibrations and find little difference in results (see Appendices S1 and S2). Due to constraints on the number of iButtons and therefore *Anolis* models available, models were not always set out at the same time as lizard abundance surveys were undertaken. However, this is unlikely to have influenced our results as the mean air temperature recorded during lizard surveys was highly correlated with the mean air temperature of the dates models were in situ (*r* = 0.85; *p* = <0.001; Figure S2.6).

#### 2.3.2 Thermal preference

We measured thermal preference for eight males and eight females of *A. bicaorum,* taken from different forest environments. Following Battles and Kolbe (2018), each individual was placed at the center of a thermal gradient (150 cm × 15 cm × 25 cm), heated by a heat lamp at one end and cooled by ice packs at the other, to obtain a gradient from approximately 10°C to 45°C. A thermocouple was inserted into the cloaca and secured with removable adhesive tape to the base of the tail. Animals were permitted to move freely within the gradient and select their preferred temperature. After a 10‐min adjustment period, internal temperatures were logged every 10 s by a data‐logger attached to the thermocouple for a total of 60 min without disturbance by observers. We then calculated the thermal preference range (*T*_pref_) by finding the central 50% of body temperatures of each animal and averaging the 25th and 75th temperature quantiles across individuals. One individual, an adult male, was excluded from subsequent calculations because it behaved unusually by not moving from the cold end of the gradient for the entire trial, despite a substantial drop in body temperature well below ambient. The duration of our thermal gradient experiments was shorter than is common in the literature (e.g., Battles & Kolbe, 2018) as a consistent temperature gradient could not be maintained for longer in field laboratory conditions and thus must be interpreted as indicative, but not definitive, measures of *T*_pref_. We examined whether *T*_pref_ estimates were affected by sampling interval by recomputing the *T*_pref_ range using 1‐min and 5‐min sampling intervals. Measures of the mean *T*_pref_ range were consistent across different sampling intervals (see Table S2.4).

#### 2.3.3 Thermal habitat quality

We calculated two indices to quantify the thermal habitat quality of each plot. The first was the percent of model hours that operative temperatures were within the *T*_pref_ range over the 36‐hr study period for each plot. The second was the total number of degrees (°C) that the models deviated from the *T*_pref_ range across all models throughout the survey period for each plot. Unlike the former, the latter includes information on the extent to which temperatures deviated both above and below the *T*_pref_ range. In *A. bicaorum's* sister species, *A*. *lemurinus*, temperatures above preferred temperature range were found to have a greater impact on lizard performance than temperatures below the range (Logan et al., 2015). Therefore, along with the total deviation, we also calculated the deviation above *T*_pref_ and the deviation below *T*_pref_ separately.

### 2.4 Structural habitat suitability

We quantified perch availability, a measure of structural microhabitat quality, by counting the number of tree trunks and palm stems within each plot. We focused on tree trunks and palm stems as we observed *A. bicaorum* almost exclusively on trunks and palm stems during microhabitat surveys, rather than on higher branches or on the ground. Where plots included fence posts, we included these in our measure of perch number. One plot had a small outbuilding, which we did not include in the measure of perch availability. As an alternative measurement of structural habitat availability, we also calculated plot basal area, a measure of stand density, by measuring each stem's diameter (including fence posts) at breast height (DBH) and using the equation, Basal Area = ∑π (DBH/2)^2^, across all tree trunks, palm stems and fence posts in the plot.

### 2.5 Prey availability

Prey availability was measured using arthropod biomass (g) from a combination of leaf litter sieving and sweep net samples taken in each plot. For sweep netting, we sampled arthropods along two diagonal transects across each plot. We sampled for five minutes along each transect. We sieved leaf litter at five locations throughout each plot: the central point of the two diagonal transects and then halfway along each transect line from the center of the plot out to the corners. All captured arthropods were placed whole in RNAlater solution for another study, then dried and weighed. No RNA extraction took place before biomass calculation. As an alternative to biomass, we identified individuals to family and calculated Simpson's and Shannon's diversity for each plot. Sweep net and leaf litter samples were combined for plot‐level analyses.

### 2.6 Leaf area index

We measured mean leaf area index (LAI) in each plot using an Accupar LP80 ceptometer. LAI is the one‐sided area of leaves per unit ground area and is a measure of canopy density; it is expected to influence thermal environment via the interception of solar radiation (Algar et al., 2018; Campbell & Norman, 1998). Ten measurements for below‐canopy photosynthetically active radiation (PAR) were taken every two meters along two diagonal transects, running from each corner of the plot. To obtain mean above‐canopy PAR, ten measurements were taken in full sunlight before and a further ten measurements were taken in full sunlight after sampling transects. We calculated LAI using a simplified version of the Norman–Jarvis model (1975). We then averaged all transect LAI values to give a mean LAI for each plot. The LAI equation and parameters are given in the Appendices S1 and S2.

### 2.7 Statistical analysis

We estimated lizard abundance using multinomial N‐mixture models. These flexible hierarchical models estimate abundance when captured individuals cannot be uniquely identified, and can incorporate detection variability and covariates of abundance (Fiske & Chandler, 2011). Models were fit using the unmarked package (Fiske & Chandler, 2011) in R version 3.5.3. Specifically, we used the multinomPois function, which fitted a multinomial‐Poisson mixture model (Royle, 2004). Before estimating abundance and whether it covaried with individual habitat metrics, we first evaluated the potential influence of differences in detection across plots by comparing the AICc of models that held abundance and detection probability constant across plots, and that allowed one or both to vary. As the model with varying abundance and constant detection rate had the lowest AICc (see Table S2.3), we constrained detection rate to be equal across plots for subsequent models. Next, we examined univariate relationships between *A. bicaorum* abundance and each of our habitat variables (percent of time within *T*_pref_, deviation from *T*_pref_, perch number, basal area, arthropod biomass, arthropod diversity, and LAI) by including each predictor as a covariate in a multinomial‐Poisson mixture model of abundance. We used these models to select a subset of these variables (one representing habitat structure, one prey availability, and one thermal quality) for subsequent path analysis, and we also included LAI as the sole measure for canopy cover. Before fitting, we standardized all predictors to have a mean of zero and standard deviation of one to allow for comparison among variables with different units. Pseudo‐*R* ^2^ values were calculated for each of the models using the *modSel* function within the unmarked package (Fiske & Chandler, 2011).

We used path analysis to evaluate the relative strength of direct and indirect effects on abundance. As we could not estimate indirect paths within a single multinomial‐Poisson mixture model, we estimated abundance for the path analysis from a multinomial‐Poisson mixture model that included no environmental covariates, held detection rate constant, and permitted abundance to vary by plot. We included the resulting abundance estimates, and log‐transformed to help meet linearity assumptions, as the response variable in our path analysis, which included all possible links between exogenous and endogenous variables. Path analysis was carried out using the lavaan (Rosseel, 2012) and semPlot (Epskamp, 2015) packages.

## 3 RESULTS

### 3.1 Variation between plots

Abundance estimates for *A. bicaorum* varied from 1 to 20 individuals across plots, with a mean abundance of 7.07 ± 2.4. *T*_pref_ for *A. bicaorum* was (mean ± *SE*) 25.4 ± 1.56°C to 28.0 ± 1.44°C. Summaries for all niche measures are given in Table 1. Data for individual plots can be seen in Table S2.2. Abundance was not correlated with mean daily air temperature (measured in the shade 1.5 m height; *r* = −.22, *p* = .46, Figure S2.11), nor was it related to survey date (*r*_s _= −.07, *p* =. 82, Figure S2.12), suggesting our results are not confounded by weather differences between days or as the field season progressed.

**TABLE 1:** Summary of structural and thermal habitat, and prey availability across 13 forest plots on Utila, Honduras

Variable	Minimum	Maximum	Mean	*SE*
Time in *T*_pref_ (%)	6.34	47.91	30.29	4.15
Sum of deviation from *T*_pref_ (^o^C)	24.4	102.15	50.35	6.69
Deviation above *T*_pref_ (^o^C)	8.95	102.15	40.73	7.21
Deviation below *T*_pref_ (^o^C)	0	40.00	9.62	4.03
Number of perches	17	232	74.38	16.18
Basal area (m^2^)	0.40	6.35	1.90	0.43
Arthropod diversity (Shannon)	0.91	1.91	1.68	0.08
Arthropod biomass (g)	0.2	2.09	1.07	0.14
LAI	0.57	3.97	2.62	0.30

### 3.2 Multinomial‐Poisson mixture models

**FIGURE 2:**
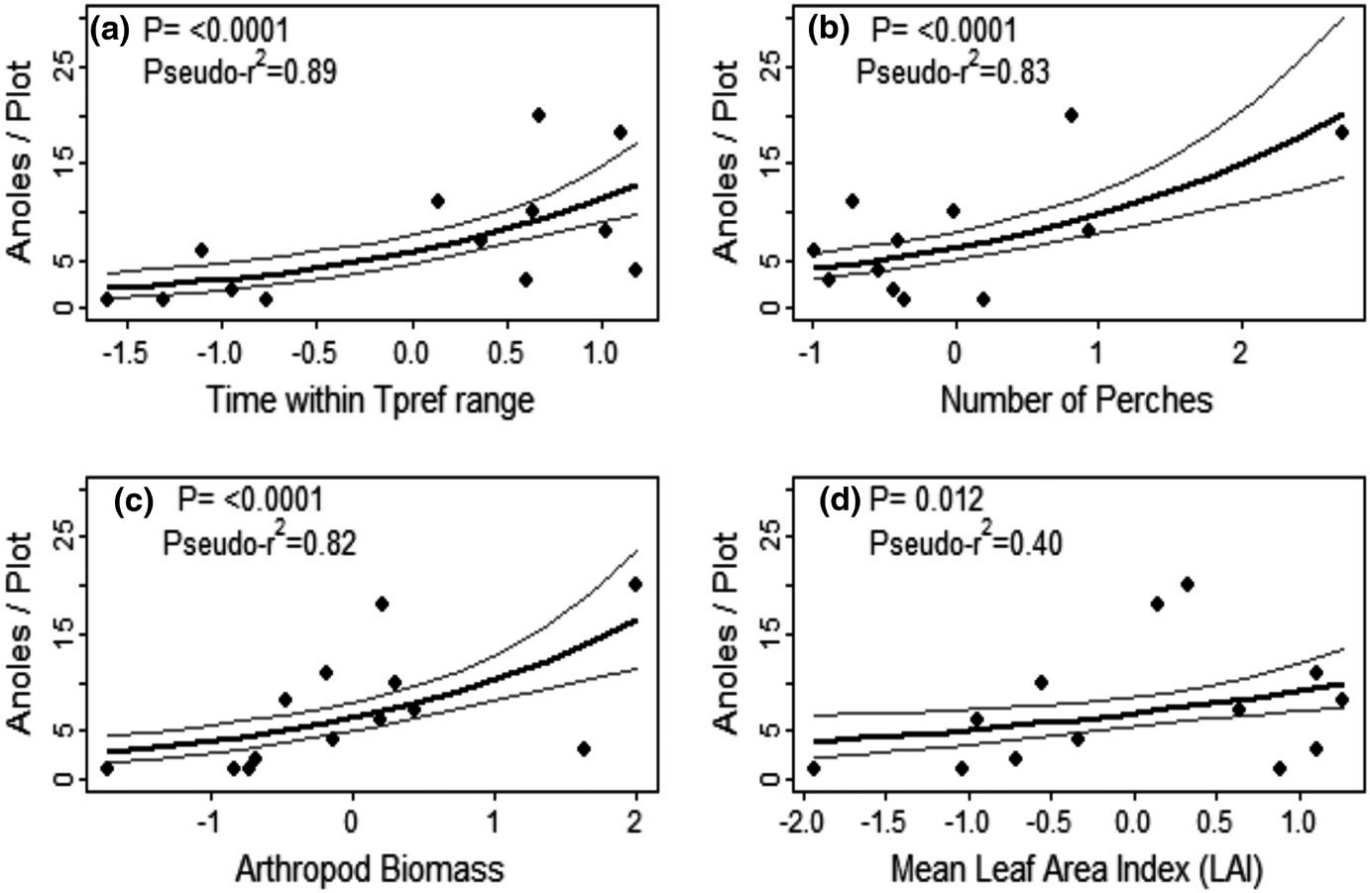
Relationships between *Anolis bicaorum* abundance and individual niche metrics in forest plots across Utila, Honduras. Relationships were estimated using multinomial‐Poisson mixture models with a constant detection rate across plots. All variables are scaled to a mean of zero and unit variance; (a) reflects thermal habitat quality, (b) reflects structural habitat quality, (c) reflects prey availability, and (d) reflects canopy cover

Abundance varied significantly with all measures of thermal habitat quality. The percentage of time each plot was within the *T*_pref_ range (Figure 2a, pseudo‐*r* ^2 ^= .89, *p =* 1.88 × 10^–6^), and the total sum of deviation of each plot (°C) from the *T*_pref_ range (pseudo‐*r* ^2^= .79, *p =* 5.17 × 10^–5^) was slightly more strongly related to abundance than the total sum of deviation above the *T*_pref_ range (pseudo‐*r* ^2 ^= .68, *p =* 4.34 × 10^–4^). The sum of deviation below the *T*_pref_ range was not significant (pseudo‐*r* ^2 ^= .02, *p =* .58). For structural microhabitat quality, the number of perches was significantly related to abundance (Figure 2b, pseudo‐*r* ^2 ^= .83, *p =* 1.92 × 10^–7^), but plot basal area was not (pseudo‐*r* ^2 ^= .03, *p =*. 52). LAI was significantly related to abundance (Figure 2d, pseudo‐*r* ^2 ^= .40, *p* = .012). Arthropod diversity (Shannon index) was not significantly related to abundance (pseudo‐*r* ^2 ^= .06, *p* = .38); using Simpson's index instead did not alter this result (pseudo‐*r* ^2 ^= .01, *p =* .69). The relationship between abundance and arthropod biomass was significant (Figure 2c, pseudo‐*r* ^2^ = .82, *p* = 1.61 × 10^–6^).

### 3.3 Path analysis

**FIGURE 3:**
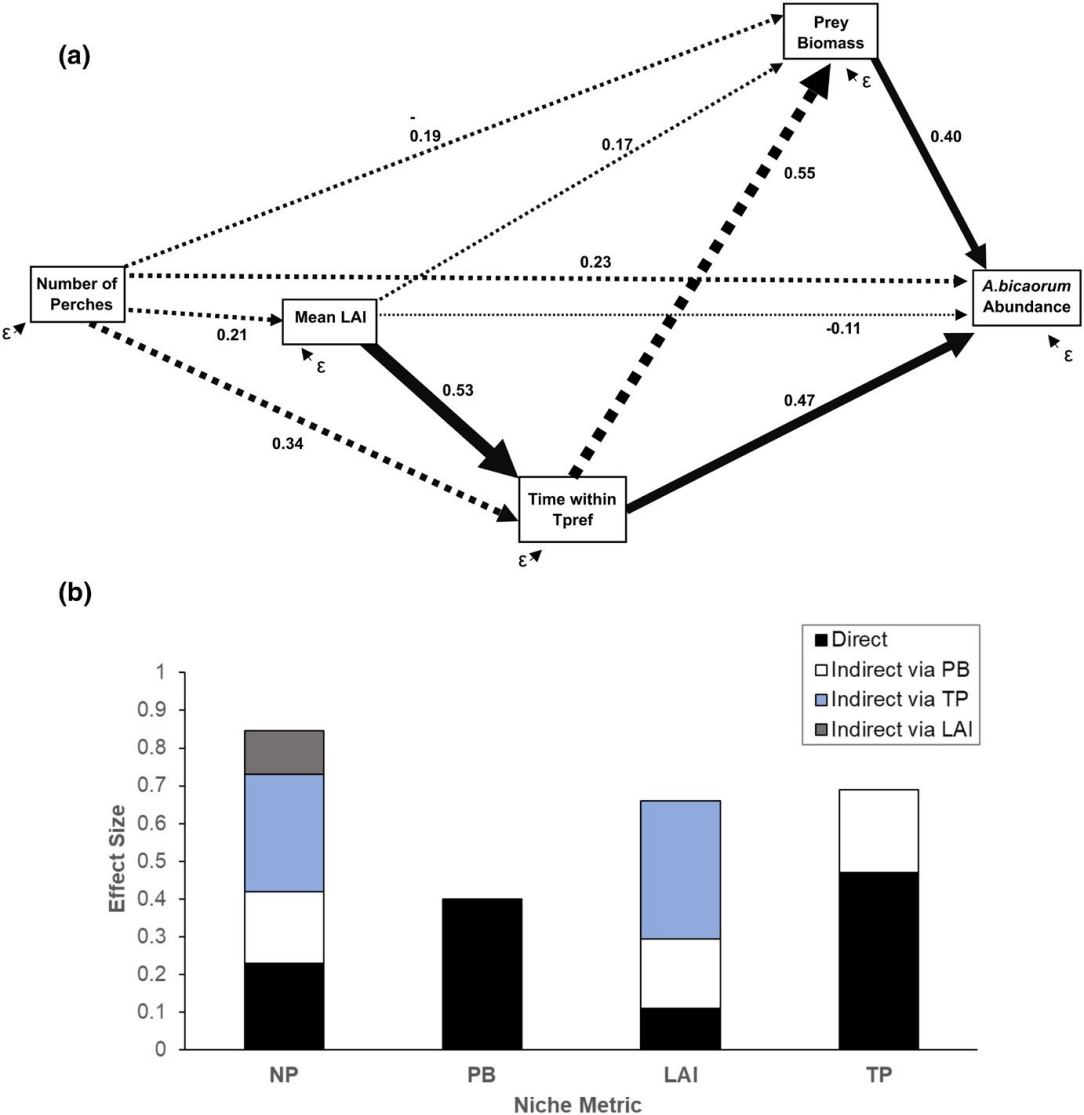
Direct and indirect effects of niche axes on *A. bicaorum* abundance. (a) Values are standardized path coefficients; line width is proportional to the strength of the effect, and solid lines indicate statistically significant pathways. ε, unexplained variation. (b) The total effects of covariates on abundance. LAI, mean leaf area index; NP, number of perches; PB, prey biomass; TP, time within *T*_pref_ range

Prey biomass and time within *T*_pref_ had the largest direct effects on *A. bicaorum* abundance (standardize coefficients: 0.40 and 0.47, respectively; Figure 3a). The path coefficient between prey biomass and abundance was significant (*p* = .049; Table 2), while the coefficient of the time within *T*_pref_ and abundance had a *p* of .055 (Table 2). LAI and number of perches had direct effects of smaller magnitude on abundance and neither were significant (Figure 3b, Table 2). Time within *T*_pref_ also had a large effect on prey biomass (*p* = .074), leading to an additional, substantial indirect effect on *A. bicaorum* abundance (Figure 3b). While LAI had little direct effect on *A. bicaorum* abundance, or on prey biomass, it had strong indirect effects through its influence on time within *T*_pref_. Number of perches had a substantial overall effect on *A.bicaorum* abundance, reflected by the large number of paths with relatively small effects, none of which were significant (Table 2, Figure 3a).

**TABLE 2:** Results of the path analysis looking at indirect and direct effects, and relationships between multiple niche axes on *A. bicaorum* abundance, in 13 forest plots on Utila, Honduras

Pathway	Estimate (±*SE*)	*Z*	*p*‐Value	Std.all
*A. bicaorum* abundance ~				
Number of perches	0.25 ± 0.20	1.25	.211	0.23
Prey biomass	0.43 ± 0.22	1.97	.049	0.40
Time within *T*_pref_	0.51 ± 0.27	1.92	.055	0.47
Mean LAI	−0.11 ± 0.22	−0.52	.609	−0.11
Time within *T*_pref_ ~				
Mean LAI	0. 53 ± 0.21	2.58	.010	0.53
Number of perches	0.34 ± 0.21	1.66	.097	0.34
Mean LAI ~				
Number of perches	0.21 ± 0.27	0.76	.449	0.21
Prey Biomass ~				
Time within *T*_pref_	0.55 ± 0.30	1.79	.074	0.55
Number of perches	−0.19 ± 0.25	−0.74	.457	−0.19
Mean LAI	0.17 ± 0.28	0.61	.545	0.17

Abbreviation: Std.all, standardized coefficients.

## 4 DISCUSSION

Classic and modern niche theory states that organisms are affected by multiple abiotic and biotic factors along multiple niche axes (Chase & Leibold, 2003). Disentangling these effects is challenging because (a) environmental changes induce change in multiple factors at once, and (b) factors are interconnected and can mediate each other's effects. Thus, we still do not have a full understanding of which niche axis exerts most pressure on abundance and the extent to which these niche axes exert direct and indirect effects. Here, we found that prey biomass and thermal habitat quality exerted the strongest direct control on the abundance of the endemic anole, *A*. *bicaorum*, on the island of Utila. However, thermal quality also had a strong indirect effect on anole abundance, mediated by prey biomass. Thermal habitat quality, in turn, was determined primarily by canopy density (LAI), which blocks incoming solar radiation, lowering operative temperatures (Algar et al., 2018) and creating heterogeneity for behavioral thermoregulation (Sears et al., 2016). Together, these results reveal the complex feedback among physical and biotic selection and highlight the importance of considering direct and indirect controls on abundance of species across habitat gradients.

The direct relationship between prey biomass and abundance is consistent with theory predicting that more food, that is, higher biomass, supports higher numbers of individuals (De Omena et al., 2019; Hatton et al., 2015). Higher food availability may also affect population dynamics and intraspecific competition. For example, more food may lead to improved body condition and energy storage within individuals, allowing for greater investment in reproduction and increased fecundity (Orrell et al., 2004). Our results also suggest that prey abundance, rather than prey diversity, is more important for maintaining population size. Many anoles are opportunistic predators (Losos, 2009), and natural history observations suggest that *A. bicaorum,* like many other anole species, is also an opportunistic predator and arthropod generalist (Brown, Maryon & Lonsdale, 2017; Köhler, 1996), although there is a lack of quantitative diet data for this species. Given its likely generalist diet, the diversity of prey taxa available should have little effect on the available resource base, which is consistent with our results. Although deforested tropical habitats often harbor reduced diversity, those species that do persist can often achieve high abundance (Foster et al., 2011), which could limit abundance declines of anoles and other generalist predators. However, we found no evidence for such compensatory dynamics here. Instead, more disturbed, built‐up areas had lower prey biomass and reduced *A. bicaorum* abundance—likely because of the reduced tree cover degrading the thermal quality of these environments. This is consistent with findings from larger urban areas, where consistent declines in abundance of multiple insect taxa have been documented (Piano et al., 2020). Thus, at least on Utila, even if some arthropod taxa benefit from disturbance leading to a loss of canopy cover, these increases are insufficient to counter overall declines in arthropod biomass, which in turn limit abundance at higher trophic levels, effects that could be further intensified by climate change (Lister & Garcia, 2018, but see Willig et al., 2019; Lister & Garcia, 2019).

Thermal habitat quality has pervasive effects on ectotherms, including physiology and behavior, which can scale to influence population dynamics (Diaz, 1997; Sinervo et al., 2010). As predicted, we found a positive association between the duration that operative temperature was within *A. bicaorum*'s *T*_pref_ and its abundance—although the *p*‐value of this relationship in the path analysis was just above .05. Individuals within their preferred temperature range for longer benefit from an increase in activity time (Gunderson & Leal, 2016), which allows increased utilization of available resources (Gvoždík, 2002) and can increase anole persistence in natural and human‐modified environments (Battles & Kolbe, 2018). Restriction of activity time, via thermal stress, can limit ectotherms' ability to effectively obtain resources, avoid predation, withstand pathogens, and reproduce effectively, leading to population declines and, ultimately, extinction (Huey et al., 2009; Sinervo et al., 2010). Our results suggest that in habitats of high thermal quality, *A. bicaorum* individuals are able to exploit longer activity times in thermally suitable plots and incur lower costs of thermoregulation. Explicitly testing this mechanism will require data on thermoregulatory efficiency of individuals across habitat types. A caveat to our results remains, however, as our estimates of *T*_pref_ in *A. bicaorum* were measured for a relatively short duration in field laboratory conditions and improved measures of *T*_pref_ are needed, including increased understanding of plastic and adaptive variation among populations.

Our results show that thermal and prey availability are not alternative controls on abundance. Rather, they are interconnected. In addition to its direct effect, thermal habitat quality had an indirect effect on *A. bicaorum* abundance, mediated by prey biomass. As arthropods are also ectotherms, they too will be affected by temperature, and their abundance is also vulnerable to warming (Lister & Garcia, 2018, but see Willig et al., 2019, Lister & Garcia, 2019). While our measure of thermal quality was focused on *A. bicaorum*, it also captured variation in prey biomass, indicating alignment in thermal niches among predators and their prey. Thus, in areas of higher thermal quality, not only do anoles have more time for foraging, but there is also more food available, providing additional benefits of thermal habitat quality that extend beyond a species thermal performance. The corollary of this is that declines in thermal habitat quality will have greater negative effects than expected solely based on a species' thermal niche. Models to predict vulnerability of ectotherms to future warming tend to focus on direct effects on activity time, thermal safety margins, and thermoregulation (e.g., Sinervo et al., 2010; Sunday et al., 2014). Our results suggest that such models may actually underestimate risks and that warming impacts may actually be magnified due to thermally induced changes in food availability, highlighting the need for greater focus on direct and indirect effects of temperature change (Duclos et al., 2019; Kearney et al., 2013).

Thermal habitat quality was primarily controlled by canopy density. Canopy cover influences microclimate in multiple ways including reducing incoming solar radiation (Campbell & Norman, 1998), which in turn lowers operative and body temperatures ectotherms (Algar et al., 2018; Kearney et al., 2009). This advantages cool‐adapted species such as *A. bicaorum* (Logan et al., 2013) and, our results reveal, their food resources as well. When overall effects are considered, LAI had a strong effect on *A. bicaorum* abundance, despite having a small direct effect. Instead, it had strong indirect effects mediated by thermal quality and, subsequently, prey biomass. While we focused on mean LAI, canopy cover may have even stronger effects than measured here as canopy heterogeneity can generate patchy thermal environments that reduce the cost of behavioral thermoregulation (Sears et al., 2016). LAI, in turn, was mildly influenced by the number of perches (stems) in a plot. Perch number, essentially stem density, had relatively weaker overall effects on abundance than LAI, and no individual paths were significant.

On Utila, personal observations suggest that human disturbance in proximity to Utila Town is the key driver of canopy variation, with clearing for housing projects ongoing, although other factors, such as variation in elevation and proximity to the coast, may also play a role. Our results highlight the pervasiveness of canopy cover for mediating ecological dynamics at higher trophic levels, not only primarily through influencing the thermal landscape (sensu Nowakowski et al., 2018) but also indirectly through mediating trophic interactions. Lastly, these findings demonstrate the importance of maintaining canopy cover and structure to maximize thermal habitat quality for cool‐adapted (Battles & Kolbe, 2018) and their prey (Lister & Garcia, 2018).

While we have identified a key role for resource availability in directly controlling anole abundance, alongside thermal environment, other biotic interactions, not examined here, may also play a role. Island anole populations are generally thought to be strongly influenced by predators, with several experiments showing substantial predator effects on anole niche dynamics and density (Pringle et al., 2019; Schoener et al., 2002). As we were not able to measure predation pressure on *A. bicaorum,* we cannot discount the possibility that predators are exerting top‐down effects on abundance, in addition to the bottom‐up effects of prey biomass. Nor can we determine the potential agonistic interactions of parasites on anole populations (Bonneaud et al., 2017). Competitive interactions could also limit abundance, although the only putative congeneric competitor, *Anolis utilensis,* is much rarer and perches much higher than *A. bicaorum* (Brown, Maryon, Van den Berg, 2017). The recently introduced brown anole, *A. sagrei*, could also have an effect on *A*. *bicaorum*'s abundance in the future, but currently, it is restricted to Utila town, where *A. bicaorum* is not found.

We have demonstrated the interconnectedness of abiotic and biotic components that determine habitat quality and animal abundance. Rather than identifying a single strong control on abundance, we found key abiotic factors (canopy cover and thermal environment) affect abundance through multiple pathways and have effects that are mediated by biotic interactions and the niche of the focal species. In particular, our results suggest alignment of thermal niches across multiple trophic levels results in strong indirect effects of thermal environment on anole abundance. Losses of thermal habitat quality, particularly due to canopy loss, may thus have greater effects than appreciated when only direct effects are considered.

## CONFLICT OF INTEREST

No conflict of interest is associated with this work.

## AUTHOR CONTRIBUTIONS

**Emma A. Higgins:** Conceptualization (equal); Data curation (equal); Formal analysis (lead); Funding acquisition (supporting); Investigation (equal); Methodology (equal); Project administration (equal); Resources (equal); Visualization (equal); Writing‐original draft (lead); Writing‐review & editing (equal). **Doreen S. Boyd:** Conceptualization (equal); Formal analysis (supporting); Funding acquisition (equal); Investigation (equal); Methodology (equal); Project administration (equal); Resources (equal); Supervision (equal); Visualization (equal); Writing‐original draft (supporting); Writing‐review & editing (equal). **Tom W. Brown:** Data curation (equal); Methodology (equal); Resources (equal); Writing‐original draft (supporting); Writing‐review & editing (equal). **Sarah C. Owen:** Conceptualization (equal); Data curation (equal); Methodology (equal); Writing‐original draft (supporting); Writing‐review & editing (equal). **Adam C. Algar:** Conceptualization (equal); Data curation (equal); Formal analysis (supporting); Funding acquisition (equal); Methodology (equal); Project administration (equal); Resources (equal); Supervision (equal); Writing‐original draft (lead); Writing‐review & editing (equal).

## PERMITS

We thank the Instituto Nacional De Conservacion y Desarrollo Forestal Areas Protegidas y Vida Silvestre (ICF) for providing relevant research permits (Permits No. DE‐MP‐054‐2017 & DE‐MP‐006‐2020). All procedures were approved by the University of Nottingham's Animal Welfare & Ethical Review Body (AWERB; approval reference no. 014).

## Supporting information

Appendix S1‐S2

## Data Availability

Data will be available from the Dryad Digital Repository following publication of the article: https://doi.org/10.5061/dryad.5mkkwh76b.
