# Supplementary material for: Disentangling controls on animal abundance: Prey availability, thermal habitat, and microhabitat structure
Source: Ecol Evol. 2021 Jul 24;11(16):11414–24. doi: 10.1002/ece3.7930 (PMC8366856; doi:10.1002/ece3.7930)
Supplement: Supplementary file 1 — Appendix S1‐S2 [file ECE3-11-11414-s001.docx]

**`SUPPORTING INFORMATION**

**Disentangling controls on animal abundance: prey availability mediates effects of thermal habitat and microhabitat structure**

**APPENDIX S1: SUPPLEMENTARY METHODS**

**3D *Anolis* model creation**

To measure operative temperature in different microhabitats at each plot, morphologically accurate 3D-printed models of *Anolis* lizards with iButtons were set up in each plot (Figure S1.1). To create the 3D model, an EinScan-S (Shining 3D) 3D Desktop Scanner was used to scan a male *Anolis sagrei* specimen provided by the Natural History Museum, London. Note that specimens of the other focus species (*Anolis bicaorum*) were not present in the collection, therefore models were modelled for a large male *A.sagrei,* which is similar in size to *A.bicaorum* (See Table S2.1). The specimen used (series no. 1938.10.4.8-79) was complete and in good condition, the collection location of the specimen was noted as Swan Island, Honduras (coordinates N17^o^ 24', W83^o^ 54') with a collection date of 14^th^ November 1937. The specimen was placed on the turntable where the EinScan-S used white light scanning to generate a 3D point cloud of the specimen, turntable rotation steps was set at 30 to increase the level of detail captured. The morphometric measurements of the specimen used can be seen in Table S1.1, all measurements refer to the right side of the anole. The three dimensional stereolithography (STL) point cloud file was imported into TinkerCAD editing software where a well was inserted to hold the iButtons. Note that due to limitations with the scanner not picking up areas of the feet and tail (due to them being smaller than 1mm), parts of the tail and the feet had to be reconstructed. Models were prepared for printing in FlashPrint 3D slicing software and printed hollow in PLA plastic using a Flashforge Creator Pro 3D printer.

Models were painted to match the solar-absorptivity of a live *Anolis* *sagrei*. To obtain the colour in the absence of live lizards, the mean RGB value was calculated from a sample of 1000 random clips of 50 *Anolis sagrei* images. These RGB values were then compared to 27 different paint samples using an online colour-sampling tool and the most suitable paint chosen. The model colour was compared to a live *Anolis sagrei* following (Munoz *et al.,* 2014 & Muñoz and Losos, 2017) by measuring reflectivity of the head, body, and tail using an Ocean Optics USB 2000 spectrometer. Reflectivity values were recorded as percent reflectance relative to a white standard using an Ocean Optics R400 ultraviolet visible (UV-VIS) reflectance probe. Note, models were painted based on *A.sagrei* as neither live animals nor sample images for *A.bicaorum* were available in the UK prior to commencing fieldwork and suitable paint was not available on the island of Utila. However, models were thermally calibrated against *A. bicaorum* (below).

**3D Model Thermal Calibration**

We calibrated T_e_ from thermal models against a live *Anolis bicaorum* following Muñoz *et al*. (2014). We inserted a thermocouple attached to an Omega /HH806AU Multilogger data logger into a live lizard (*A.bicaorum*) and a 3D model simultaneously and exposed them to different environmental conditions. Both the lizard and the model were first placed in a cool place (icebox) until their temperatures reached around 20^o^C (and model and animal were the same temperature). The lizard and model were then moved into the sun where their temperatures were logged for approximately 15 minutes at 15 second intervals, or until the animal’s temperature reached 32^o^C. Model and lizard were then moved back into the original cool place (icebox) and their temperatures taken for a further 15 minutes. As models reached higher temperatures than the live animal, which could skew estimates of time spent within the preferred temperature range (Figure S2.4), we generated a transfer function to convert 3D model T_e_ to lizard T_e_. We modelled the change in lizard T_b_ (ΔT_b_ ) per second as function of the change in 3D model T_e_ (ΔT_e_) per second using linear regression through the origin (Figure S2.5), which had R^2^=0.74, and was statistically significant (F=271.9, df=1,94, P<1×10^-15^). We tested for curvilinearity using a quadratic regression, but the quadratic term was not significant (P=0.92, R^2^=0.74). The linear regression produced the following equation (slope ± s.e.):

$\Delta Tb = 0.41\pm0.02\times\Delta Te$ ***- Equation S1***

We used this equation to model how lizard Tb would change in each time step, assuming that T_b_ and T_e_ were equal at 6am each day, which is realistic given the lack of direct solar radiation at this time. Comparing transformed T_e_ values (using Equation S1) to the anole T_b_ during the calibration experiment, we found that using equation S1 over-corrected T_e_ (Figure S2.4). Thus, our uncalibrated T_e_ measures and over-corrected T_e_ values represent an envelope in which the true T_e_ occurs. We thus present analyses using the average of the uncalibrated and over-corrected T_e_ values in the main text (calibrated T_e_) and results using the uncalibrated and over-corrected T_e_ in this Supplementary Material. We found that the results were similar and led to the same conclusions regardless of whether calibrated, uncalibrated, or over-corrected T_e_ was used (see Table S2.5, Figures S2.7-S2.9). Thus our results are not an artefact of imperfections in T_e_ models.

**Leaf area index calculation**

A general introduction to leaf area index (LAI) and radiation transmission through plant canopies can be found in Chapter 15 of Campbell and Norman (1998). We calculated LAI using a simplified version of the Norman-Jarvis model, as presented in Decagon Devices Inc (2013):

$LAI = \frac{[ 1 - 1 2K fb - 1]ln\tau}{A(1 - 0.47fb)}$ - ***Equation S2***

where τ is the ratio of transmitted to incident photosynthetically active radiation (PAR), measured with the ceptometer. K is the extinction coefficient for the canopy, Fb is the fraction of incident PAR which is beam and is estimated by the ceptometer from incident radiation and the solar constant (Decagon Devices Inc (2013). In Equation S2, A is a function of leaf absorptivity in the PAR band (a, see below). K was modelled as a function of zenith angle (Θ), assuming a leaf angle distribution of 1.0, the default value for the AccuPAR LP-80 ceptometer. Calculations of LAI are not strongly affected by the leaf angle distribution (Decagon Devices Inc 2013).

$K=\frac{1}{2Cos\Theta}$ ***- Equation S3***

In Equation S2, A was calculated as:

A= 0.283 + 0.785a – 0.159 a^2^ ***– Equation S4***

Where a is the leaf absorptivity in the PAR band. The AccuPAR LP-80 ceptometer assumes 0.9 in LAI sampling routines (see user manual – Decagon Devices, 2013).

**References**

Campbell, G. S., and J. M. Norman. 1998. An introduction to environmental biophysics. 2nd edition. - Springer-Verlag, New York.

Muñoz, M. M. et al.. 2014. Evolutionary stasis and lability in thermal physiology in a group of tropical lizards. – Proc Royal Soc B. 281: 20132433.

Decagon Devices Inc. (2013) AccuPAR PAR/LAI Ceptometer Model LP-80 Operator’s Manual. Pullman Washington: Decagon Devices Inc, pp. 40-42.

**APPENDIX S2: SUPPLEMENTARY TABLES AND FIGURES**

**Table S2.1**: Morphometric Measurements of *Anolis sagrei* Museum Specimen

| **Feature** | **Measurement (mm)** |
| --- | --- |
| Snout to Right Femur Insertion | 58.35 |
| Humerus Length | 12.29 |
| Ulna Length | 11.28 |
| Finger IV Length | 9.81 |
| Femur Length | 16.41 |
| Tibia Length | 18.6 |
| Metatarsus length | 10.72 |
| Toe IV Length | 12.43 |

**Table S2.2:** Summaries of thermal and structural habitat and prey availability, across all 13 plots

| **Plot No.** | **Time within T_pref_**  **(%)** | **Sum of Deviation**  **from T_pref_**  **(^o^C)** | **Deviation**  **above T_pref_**  **(^o^C)** | **Deviation**  **below T_pref_**  **(^o^C)** | **No.**  **Perches** | **Basal**  **Area**  **(m^2^)** | **Arthropod**  **Diversity**  **(Shannon)** | **Arthropod**  **Biomass (g)** | **Mean LAI** |
| --- | --- | --- | --- | --- | --- | --- | --- | --- | --- |
| 1 | 45.61 | 26.42 | 15.63 | 10.78 | 129 | 2.27 | -1.91 | 0.84 | 3.97 |
| 2 | 39.36 | 28.25 | 19.3 | 8.95 | 23 | 0.67 | -1.91 | 1.90 | 3.81 |
| 3 | 35.76 | 30.26 | 20.42 | 9.84 | 51 | 1.08 | -1.79 | 1.30 | 3.30 |
| 4 | 16.19 | 65.11 | 25.11 | 40 | 49 | 1.05 | -1.63 | 0.73 | 1.87 |
| 5 | 13.74 | 79.45 | 39.45 | 40 | 17 | 1.34 | -1.89 | 1.18 | 1.62 |
| 6 | 6.34 | 77.58 | 77.58 | 0 | 54 | 1.05 | -0.91 | 0.70 | 1.53 |
| 7 | 39.87 | 45.45 | 45.45 | 0 | 74 | 1.71 | -1.68 | 1.23 | 2.04 |
| 8 | 40.25 | 24.40 | 8.95 | 15.45 | 122 | 1.86 | -1.86 | 2.09 | 2.97 |
| 9 | 18.91 | 50.05 | 50.05 | 0 | 86 | 3.10 | -1.86 | 0.65 | 3.58 |
| 10 | 10.64 | 102.15 | 102.15 | 0 | 54 | 0.40 | -1.57 | 0.20 | 0.57 |
| 11 | 46.69 | 40.47 | 40.47 | 0 | 232 | 2.76 | -1.33 | 1.18 | 2.79 |
| 12 | 47.91 | 35 | 34.94 | 0.06 | 43 | 6.35 | -1.70 | 1.01 | 2.27 |
| 13 | 32.44 | 50 | 50 | 0 | 33 | 1.18 | -1.73 | 0.98 | 3.81 |
|  |  |  |  |  |  |  |  |  |  |

**Table S2.3:**  AICc values for N-mixture models of abundance that allow abundance and or detectability of *A. bicaorum* to vary by site, or hold them constant. AICc values determined using the modSel function within the unmarked package in R.

| **Model** | **Description** | **AICc** |
| --- | --- | --- |

| M0 | Null model | 399.87 |
| --- | --- | --- |
| Mboth | Model where abundance and detectability can vary by plot | 252.64 |

| Mdet | Model where only detectability can vary by  plot | 184.03 |
| --- | --- | --- |
| MSite | Model where only abundance can vary by  plot | 147.57 |

**Table S2.4:** T_pref_ range of A.bicaorum measured at different sampling intervals of 10 seconds, 1 minute and 5 minutes.

| **Measurement Interval** | **Lower T_pref_ Range (± S.E)** | **Upper T_pref_ Range (± S.E)** |
| --- | --- | --- |

| 10 Seconds | 25.4 ± 1.56°C | 28.0 ± 1.44°C |
| --- | --- | --- |
| 1 Minute | 25.4 ± 1.53°C | 28.0 ± 1.35°C |

| 5 Minutes | 25.5 ± 1.29°C | 28.0 ± 1.27°C |
| --- | --- | --- |

**
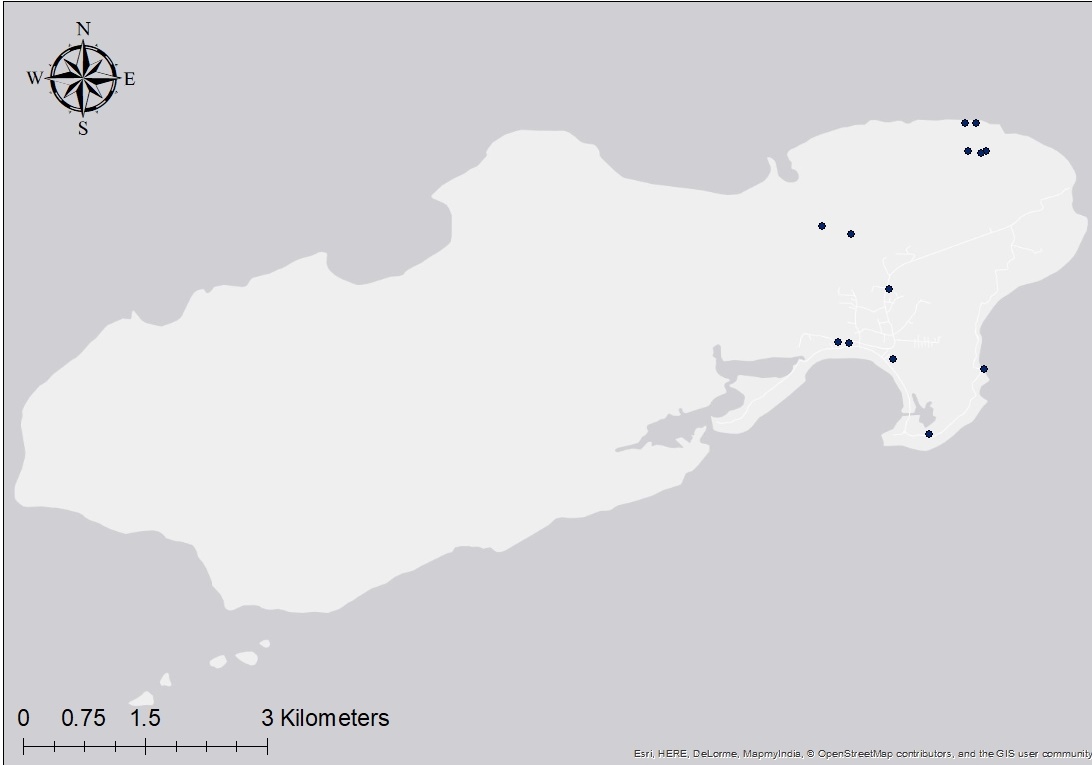

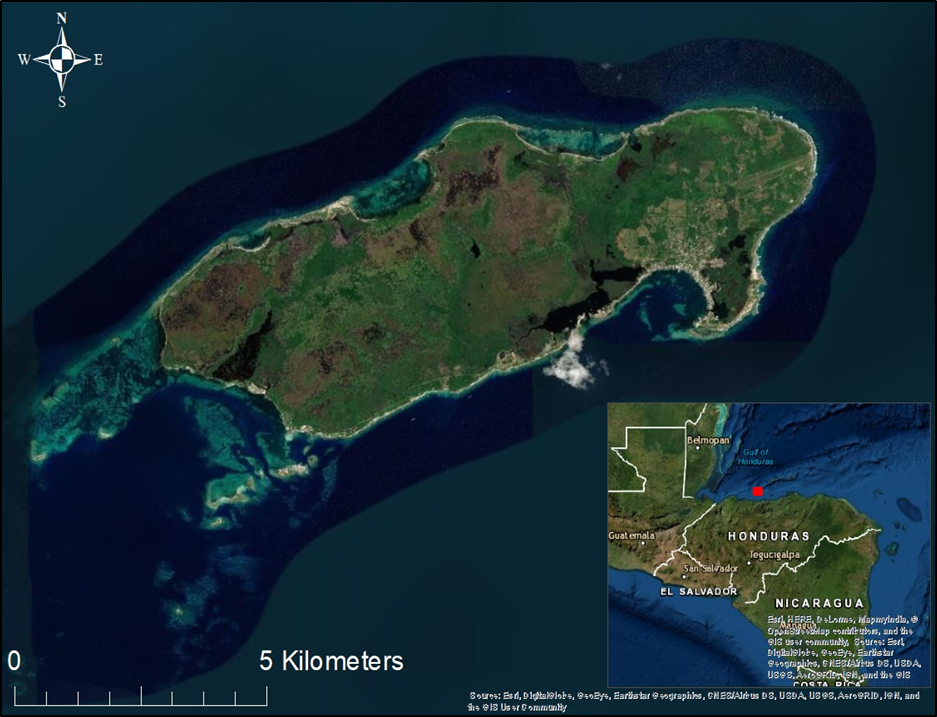
**

**Figure S2.1:** (left) Location of Utila, Isla de Bahia, Honduras, (right) location of survey plots.


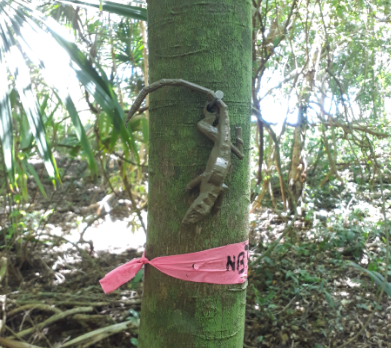


**Figure S2.2:** 3D Anolis Model


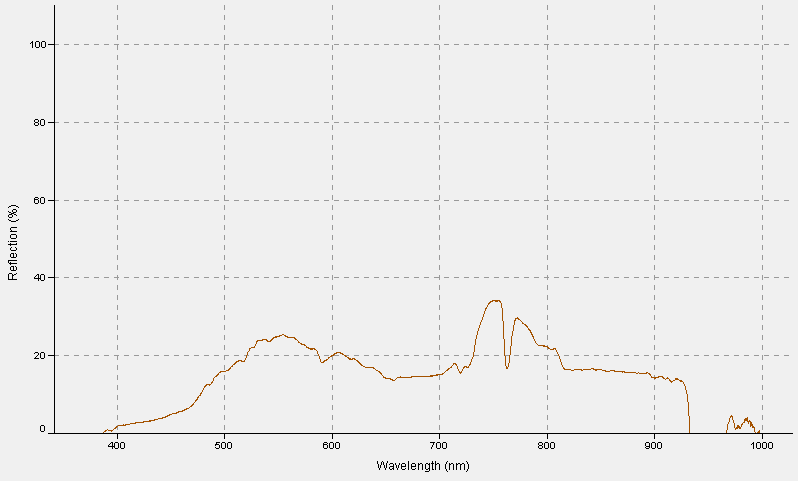

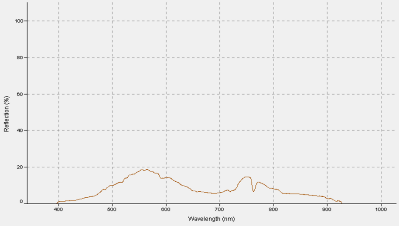


**Figure S2.3:** Reflectivity values as a percent reflectance relative to a white standard for the 3D models (top) and the live *A.sagrei* (bottom)

**Figure S2.4:** T_b_ of an adult male *Anolis bicaorum* (animal), uncalibrated, calibrated and over-corrected T_e_ over time during the calibration experiment.**
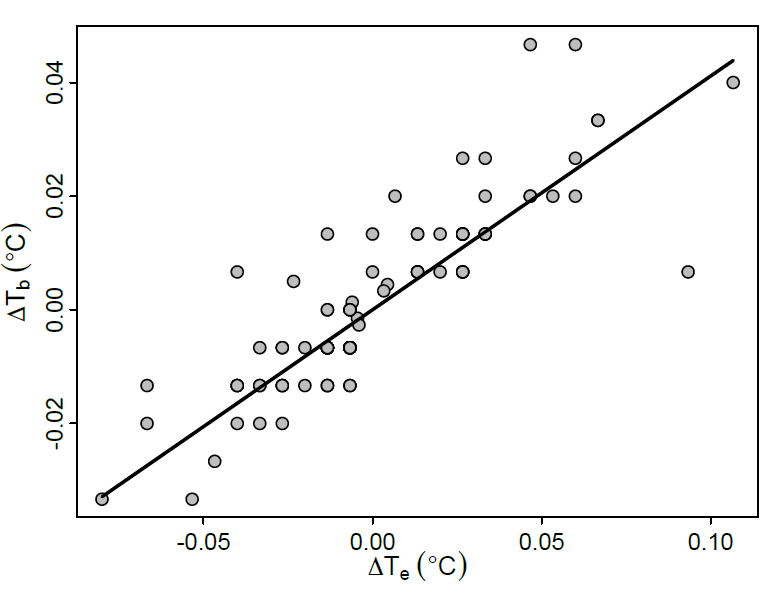
**

**Figure S2.5:** Change in lizard Tb (ΔTb ) per second as function of the change in 3D model Te (ΔTe) per second

**
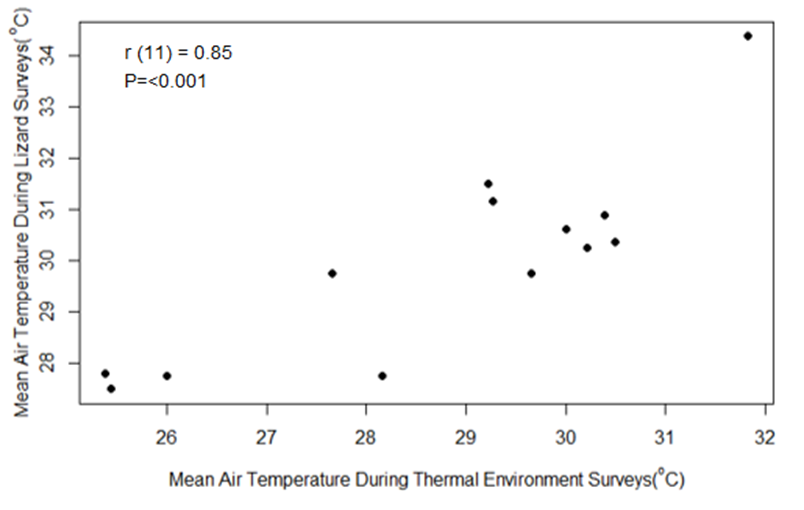
**

**Figure S2.6:** Comparison of mean air temperature recorded over all lizard surveys with the mean air temperature of the dates models were in-situ for the same times as lizard surveys (09:00, 13:00 and 17:00) across the 13 survey plots**.**


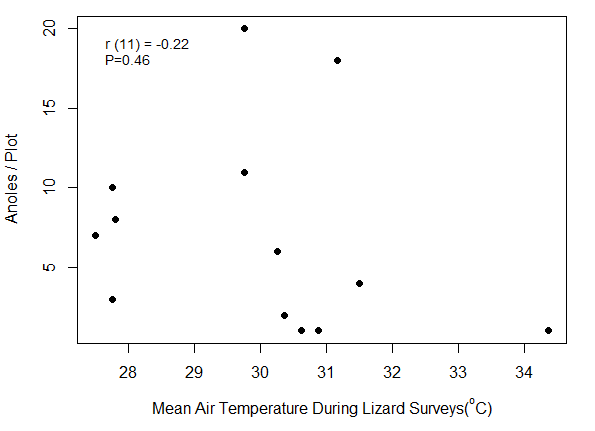


**Figure S2.7:** Comparison of anole abundance (Anoles/ Plot) with mean air temperature during lizard (abundance) surveys (^o^C).


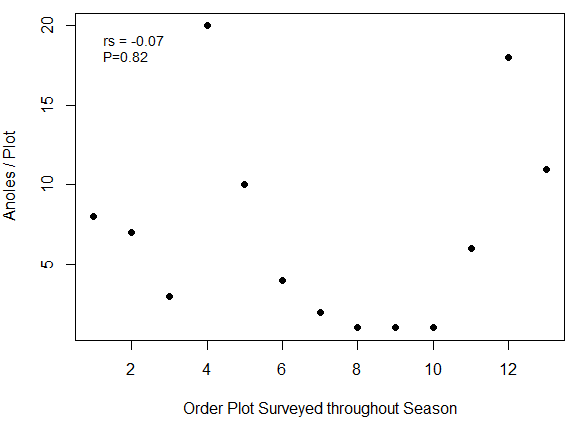


**Figure S2.8:** Comparison of anole abundance (Anoles/ Plot) with the order in which the plots were surveyed throughout the field season. Rank based on date order surveyed.

**ADDITIONAL RESULTS – CALIBRATED THERMAL MODEL DATA**


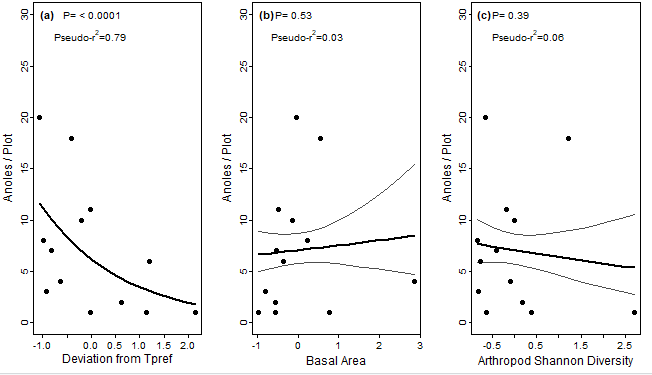


**Figure S2.9:** Relationships between *Anolis bicaorum* abundance and individual niche metrics in forest plots across Utila, Honduras, that were not included in the final analysis. Relationships were estimated using multinomial Poisson mixture models with a constant detection rate across plots. All variables are scaled to a mean of zero and unit variance, (a) deviation from T_pref_ range, (b) basal area and (e) prey diversity.

**RESULTS USING UNCALIBRATED THERMAL MODEL DATA**


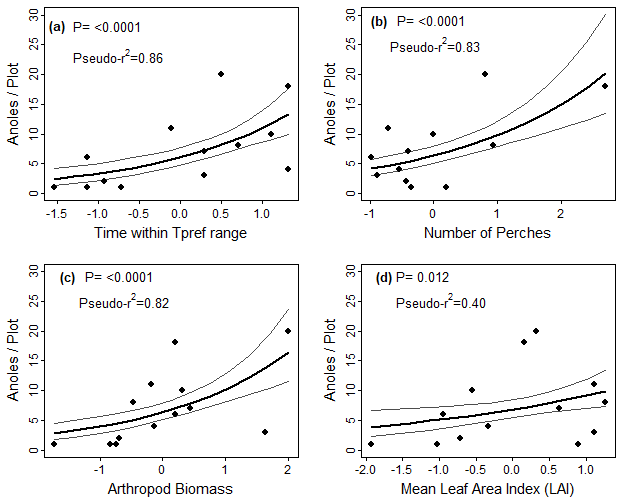


**Figure S2.10:** Relationships between *Anolis bicaorum* abundance and individual niche metrics in forest plots across Utila, Honduras, using the original uncalibrated thermal model data. Relationships were estimated using multinomial Poisson mixture models with a constant detection rate across plots. All variables are scaled to a mean of zero and unit variance, (a) reflects thermal habitat quality, (b) reflects structural habitat quality, (c) reflects prey availability and (d) canopy cover.


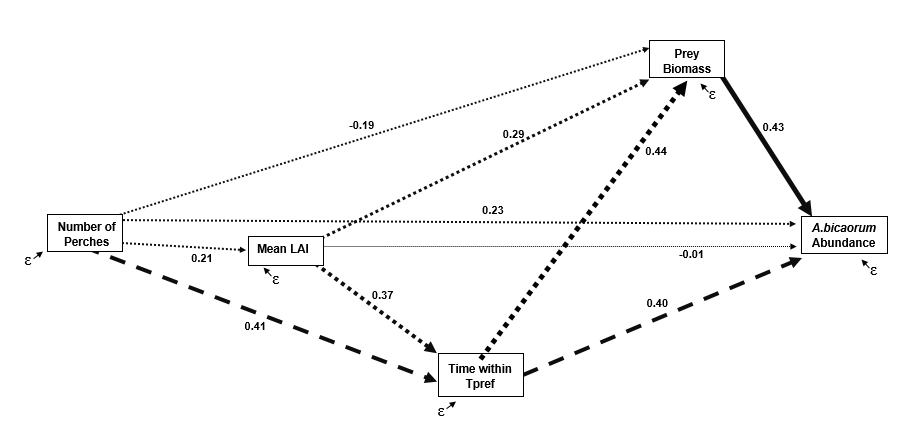
**
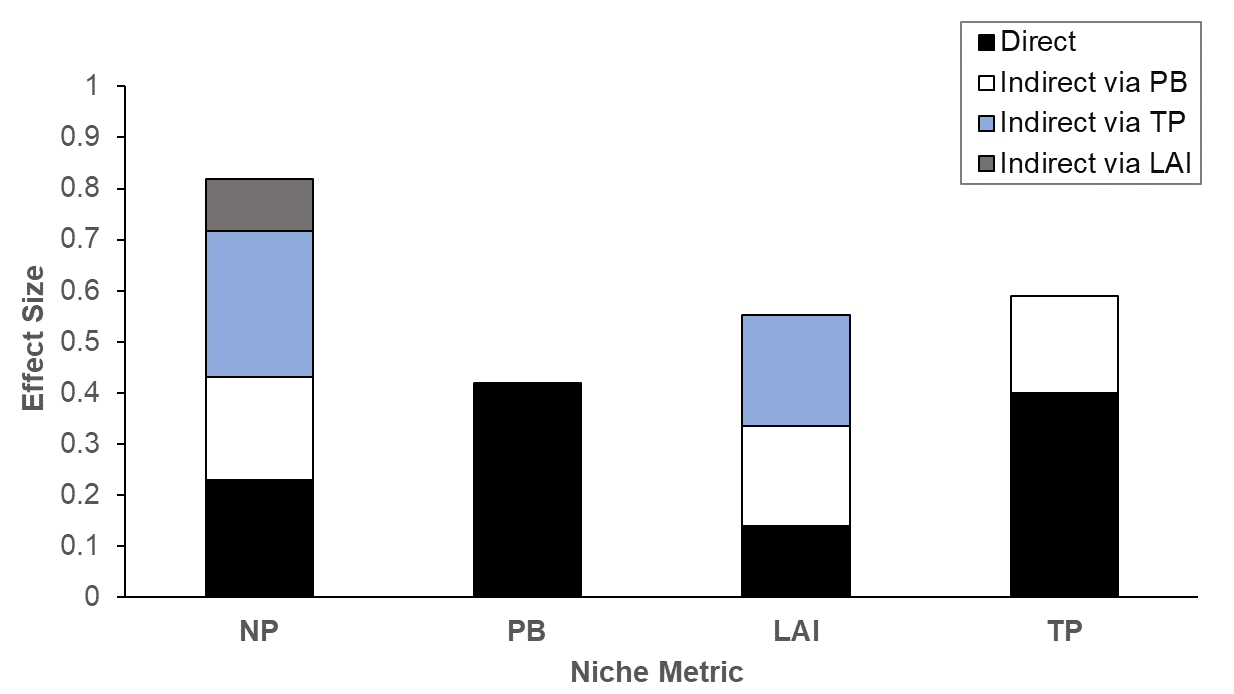
**

**(b)**

**(a)**

**Figure S2.11:** Direct and indirect effects of niche axes on *A. bicaorum* abundance using the original uncalibrated thermal model data. (a) Values are standardized path coefficients; line width is proportional to the strength of the effect, solid lines indicate P<0.05, dashed lines 0.05≥P<0.10, and dotted lines P≥0.10, and ε is unexplained variation. (b) The total effects of covariates on abundance. NP: number of perches; PB: prey biomass; LAI: mean leaf area index; TP: time within Tpref range.

**Table S2.5:** Results of the path analysis on the original uncalibrated thermal model data looking at indirect and direct effects, and relationships between, multiple niche axes on *A. bicaorum* abundance, in 13 forest plots on Utila, Honduras. Std.all, Standardised Coefficients.

| **Pathway** | **Estimate (± S.E)** | ***Z*** | ***P*-Value** | **Std.all** |
| --- | --- | --- | --- | --- |

|  |  |  |  |  |
| --- | --- | --- | --- | --- |
| ***A.bicaorum* Abundance ~** |  |  |  |  |
| Number of Perches | 0.24 ± 0.21 | 1.12 | 0.238 | 0.23 |

| Prey Biomass | 0.46 ± 0.21 | 2.14 | 0.032 | 0.43 |
| --- | --- | --- | --- | --- |
| Time within T_pref_ | 0.43 ± 0.24 | 1.78 | 0.076 | 0.40 |
| Mean LAI | -0.15 ± 0.21 | -0.75 | 0.94 | -0.01 |
|  |  |  |  |  |
| **Time within T_pref_ ~** |  |  |  |  |
| Mean LAI | 0.37 ± 0.22 | 1.64 | 0.100 | 0.37 |
| Number of Perches | 0.42 ± 0.22 | 1.86 | 0.063 | 0.42 |
|  |  |  |  |  |
| **Mean LAI ~** |  |  |  |  |
| Number of Perches | 0.21 ± 0.27 | 0.76 | 0.449 | 0.21 |
|  |  |  |  |  |
| **Prey Biomass ~** |  |  |  |  |
| Time within T_pref_ | 0.45 ± 0.27 | 1.56 | 0.119 | 0.45 |
| Number of Perches | -0.19 ± 0.26 | -0.71 | 0.476 | -0.18 |
| Mean LAI | 0. 29 ± 0.26 | 1.15 | 0.249 | 0.29 |


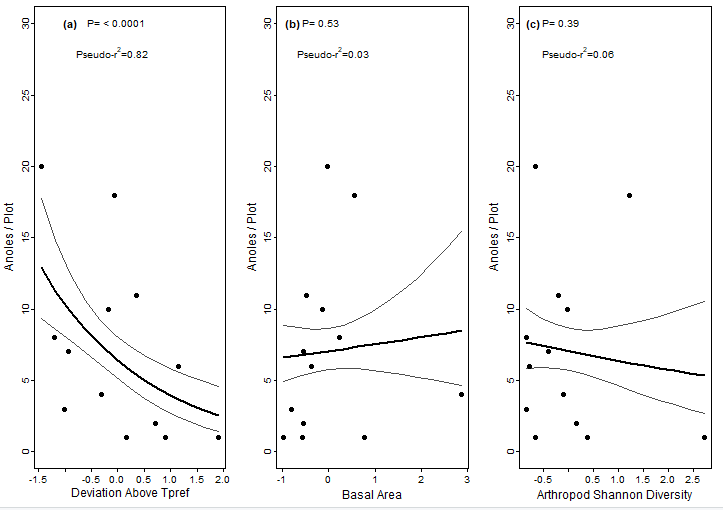


**Figure S2.12:** Relationships between *Anolis bicaorum* abundance and individual niche metrics excluded from the final analysis, using the original uncalibrated thermal model data. Relationships were estimated using multinomial Poisson mixture models with a constant detection rate across plots. All variables were scaled to a mean of zero and unit variance, (a) deviation from T_pref_ range, (b) basal area, (c) prey diversity.

**RESULTS OF OVER-CORRECTED THERMAL MODEL DATA**


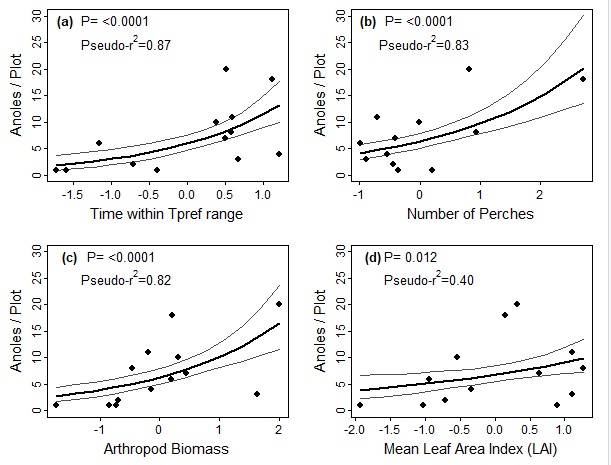


**Figure S2.13:** Relationships between *Anolis bicaorum* abundance and individual niche metrics in forest plots across Utila, Honduras for the over-corrected thermal model data. Relationships were estimated using multinomial Poisson mixture models with a constant detection rate across plots. All variables are scaled to a mean of zero and unit variance; (a) reflects thermal habitat quality, (b) reflects structural habitat quality, (c) reflects prey availability and (d) reflects canopy cover.


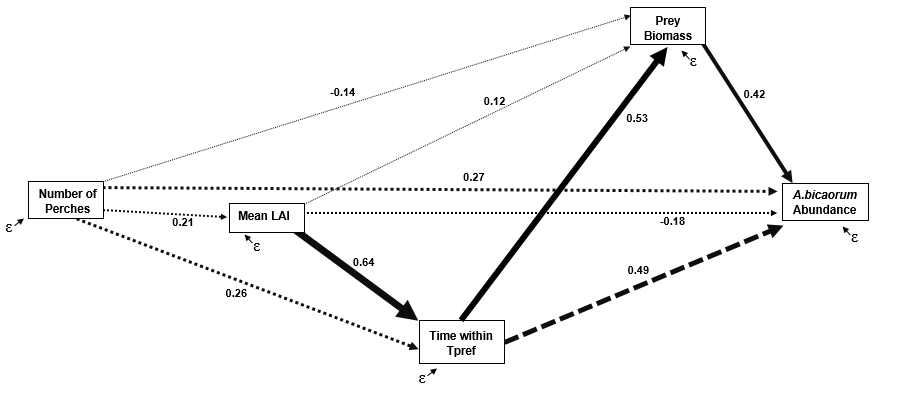


**(b)**

**(a)**


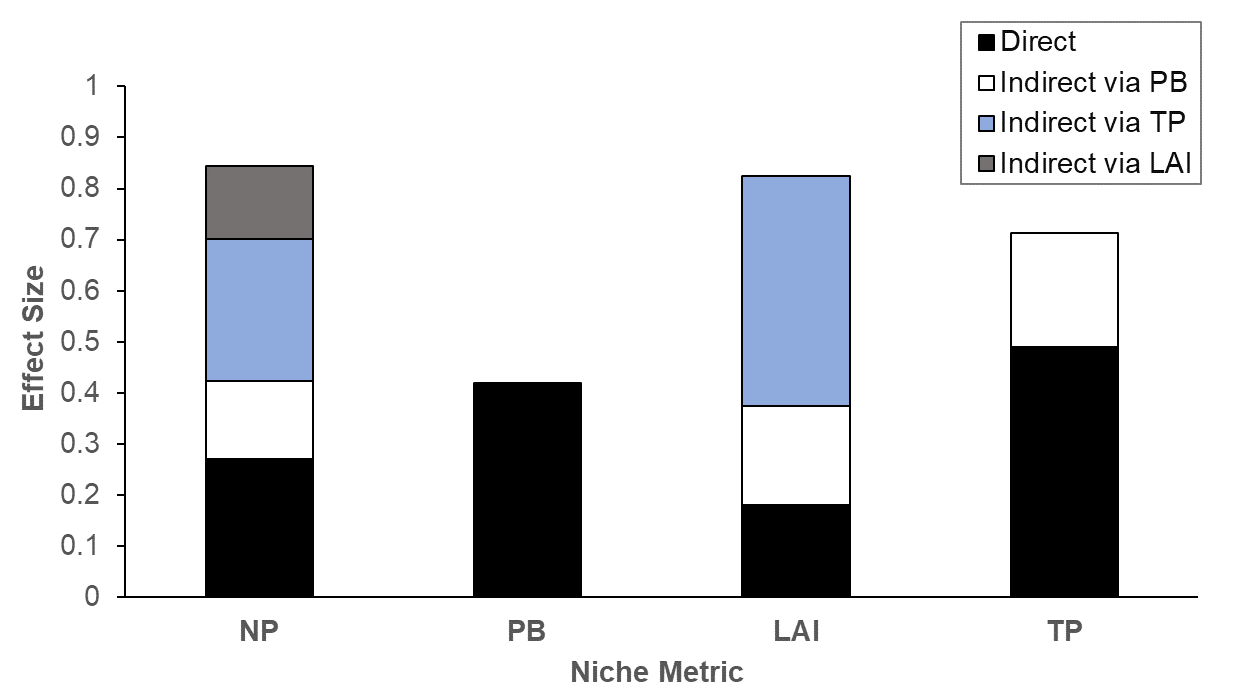


**Figure S2.14:** Direct and indirect effects of niche axes on *A. bicaorum* abundance for the over-corrected thermal model data. (a) Values are standardized path coefficients; line width is proportional to the strength of the effect, solid lines indicate P<0.05, dashed lines 0.05≥P<0.10, and dotted lines P≥0.10, and ε is unexplained variation. (b) The total effects of covariates on abundance. NP: number of perches; PB: prey biomass; LAI: mean leaf area index; TP: time within Tpref range.

**Table S2.6:** Results of the path analysis on the over-corrected thermal model data looking at indirect and direct effects, and relationships between, multiple niche axes on *A. bicaorum* abundance, in 13 forest plots on Utila, Honduras. Std.all, Standardised Coefficients.

| **Pathway** | **Estimate (± S.E)** | ***Z*** | ***P*-Value** | **Std.all** |
| --- | --- | --- | --- | --- |

|  |  |  |  |  |
| --- | --- | --- | --- | --- |
| ***A.bicaorum* Abundance ~** |  |  |  |  |
| Number of Perches | 0.29 ± 0.19 | 1.51 | 0.132 | 0.27 |

| Prey Biomass | 0.45 ± 0.21 | -0.78 | 0.035 | 0.42 |
| --- | --- | --- | --- | --- |
| Time within T_pref_ | 0.58 ± 0.28 | 1.90 | 0.058 | 0.49 |
| Mean LAI | -0.19 ± 0.24 | -0.78 | 0.434 | -0.18 |
|  |  |  |  |  |
| **Time within T_pref_ ~** |  |  |  |  |
| Mean LAI | 0.64 ± 0.19 | 3.31 | 0.001 | 0.64 |
| Number of Perches | 0.26 ± 0.19 | 1.34 | 0.184 | 0.26 |
|  |  |  |  |  |
| **Mean LAI ~** |  |  |  |  |
| Number of Perches | 0.21 ± 0.27 | 0.76 | 0.449 | 0.21 |
|  |  |  |  |  |
| **Prey Biomass ~** |  |  |  |  |
| Time within T_pref_ | 0.53 ± 0.33 | 1.60 | 0.110 | 0.53 |
| Number of Perches | -0.14 ± 0.25 | -0.55 | 0.583 | -0.14 |
| Mean LAI | 0.12 ± 0.31 | 0.38 | 0.703 | 0.12 |


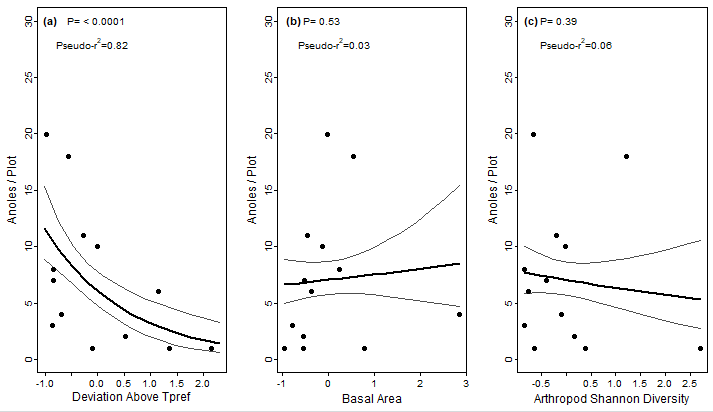


**Figure S2.15:** Relationships between *Anolis bicaorum* abundance and individual niche metrics excluded from the final analysis, using the over-corrected thermal model data. Relationships were estimated using multinomial Poisson mixture models with a constant detection rate across plots. All variables were scaled to a mean of zero and unit variance, (a) deviation from T_pref_ range, (b) basal area, (c) prey diversity.
